# Prenatal intrauterine growth restriction and risk of retinopathy of prematurity

**DOI:** 10.1038/s41598-020-74600-0

**Published:** 2020-10-16

**Authors:** Alison Chu, Yasmeen Dhindsa, Myung Shin Sim, Marie Altendahl, Irena Tsui

**Affiliations:** 1 David Geffen School of Medicine, Department of Pediatrics, Division of Neonatology and Developmental Biology, University of California Los Angeles, 10833 Le Conte Avenue, Room B2-375 MDCC, Los Angeles, CA 90095 USA; 2 David Geffen School of Medicine, Department of Medicine Statistics Core, University of California Los Angeles, Los Angeles, CA USA; 3 David Geffen School of Medicine, Department of Ophthalmology, University of California Los Angeles, Los Angeles, CA USA

**Keywords:** Medical research, Paediatric research, Risk factors, Intrauterine growth, Preterm birth, Retinopathy of prematurity, Retinal diseases, Retinopathy of prematurity

## Abstract

Low birthweight and decreased postnatal weight gain are known predictors of worse retinopathy of prematurity (ROP) but the role of prenatal growth patterns in ROP remains inconclusive. To distinguish small for gestational age (SGA) from intrauterine growth restriction (IUGR) as independent predictors of ROP, we performed a retrospective cohort study of patients who received ROP screening examinations at a level IV neonatal intensive care unit over a 7-year period. Data on IUGR and SGA status, worst stage of and need for treatment for ROP, and postnatal growth was obtained. 343 infants were included for analysis (mean gestational age = 28.6 weeks and birth weight = 1138.2 g). IUGR infants were more likely to have a worse stage of ROP and treatment-requiring ROP (both *p* < 0.0001) compared to non-IUGR infants. IUGR infants were more likely to be older at worst stage of ROP (*p* < 0.0001) and to develop postnatal growth failure (*p* = 0.01) than non-IUGR infants. Independent of postnatal growth failure status, IUGR infants had a 4–5 × increased risk of needing ROP treatment (*p* < 0.001) compared to non-IUGR infants. SGA versus appropriate for gestational age infants did not demonstrate differences in retinopathy outcomes, age at worst ROP stage, or postnatal growth failure. These findings emphasize the importance of prenatal growth on ROP development.

## Introduction

Retinopathy of prematurity (ROP) is a disorder characterized by abnormal retinal blood vessel growth in preterm infants. In some patients, if left untreated, ROP can lead to visual impairment and blindness^1^. In postnatal phase 1 of ROP, increased oxygen exposure suppresses hypoxia-inducible factor 1 alpha (Hif1α) and vascular endothelial growth factor (VEGF), contributing to vascular attenuation. In postnatal phase 2, relative local hypoxia promotes increased Hif1α and VEGF, fueling pathologic blood vessel proliferation^2^. Moreover, postnatal weight gain, as a surrogate of insulin growth factor 1 (IGF-1) levels and protective against ROP, has been suggested as an additional screening filter in an effort to reduce unnecessary eye exams^3^. However, it is possible that there is an even earlier “phase” of ROP development which occurs in utero, such that an abnormal prenatal environment may modulate risk for postnatal development of ROP.

Small for gestational age (SGA) is a term utilized to describe a neonate born below the 10th percentile of BW by age and sex^4^. SGA status does not distinguish the etiology for low BW; SGA may be constitutional low BW due inherent genetic potential or secondary to a pathologic environment in utero. IUGR, in comparison, also can result in neonates who have low BW but more specifically is characterized by growth deceleration of the fetus in utero^5^. Unfortunately, attempts to standardize how IUGR should be defined have not occurred until recently. The American College of Obstetricians and Gynecologists released published guidelines in 2019, suggesting the term “fetal growth restriction” to replace IUGR, along with a number of specific fetal growth indices in addition to estimated fetal weight, such as abdominal circumference, to help diagnose IUGR^6^. Importantly, a neonate can be SGA, IUGR, or both depending on prenatal anthropometrics and postnatal birthweight (BW). The few existing studies variably suggest that SGA status may be a risk factor for severe and treated ROP^7^, implying that prenatal growth patterns do dictate propensity for postnatal development of ROP. However, to our knowledge, there are no known studies that have evaluated intrauterine growth restriction (IUGR) separate from SGA in relation to ROP even though they are clinically distinct in terms of pathophysiology and neonatal outcomes.

IUGR status implies pathologic etiologies and oftentimes results from maternal conditions that impair blood flow and oxygen provision to the growing fetus (typically presenting as asymmetric growth restriction), such as maternal hypertensive disorders. IUGR may also result from congenital infections and genetic disorders (typically presenting as symmetric growth restriction)^4^. Although IUGR is often utilized interchangeably with SGA in clinical practice, IUGR fetuses have been shown to have worse systemic outcomes^8^. Our hypothesis, therefore, was that IUGR, not SGA, is an independent risk factor for ROP treatment.

## Results

### Study population

**Figure 1:**
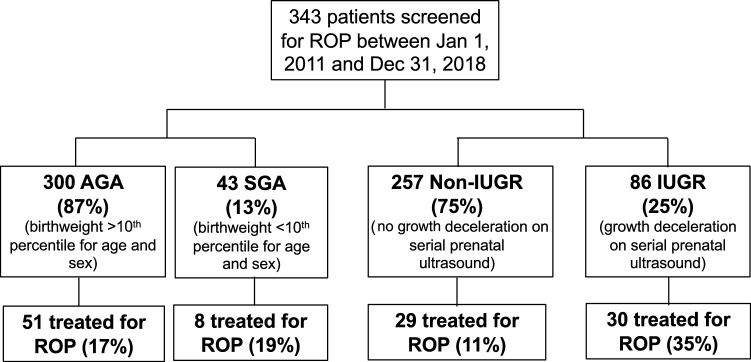
Patient population and treatment frequency. All infants were categorized as either SGA or AGA, and IUGR or not IUGR, separately.

356 infants were identified as receiving screening ROP exams and 13 infants excluded for missing data, leaving 343 infants included in our analyses. The mean GA of the population was 28.6 weeks, (range = 22–1/7 to 34–3/7 weeks; SD = 2.78). The mean BW of the population was 1138 g (range = 410–2940 g; SD = 401.1). 45% of infants were female and 55% of infants were male. 12.5% of infants (43/343) were defined as being SGA and 25.1% of infants were identified as IUGR (86/343) (Fig. 1). The demographic characteristics (GA, BW, and CGA at worst ROP exam, inborn vs outborn, and sex) for the SGA and IUGR groups are shown in Tables 1 and 2. There were more males in the SGA cohort (72.1%) than in the appropriate for gestational age (AGA) cohort (53.0% male) (*p* = 0.019, 95% CI 4.4–33.5%). There were no differences in the proportion of males in IUGR (51.2% male) vs. non-IUGR (56.8% male) infants (*p* = 0.362, 95% CI − 6.6 to 17.8%). 51.6% of the infants were outborn, and 48.4% were inborn. There were no differences in the proportion of inborn versus outborn infants in the SGA versus AGA groups (AGA: 47.7% inborn, SGA: 53.5% inborn; *p* = 0.475 by Chi-square, 95% CI − 10.0 to 22.0%), or in the IUGR versus non-IUGR groups (non-IUGR: 50.2% inborn, IUGR: 43.0%; *p* = 0.249 by Chi-square, 95% CI − 4.9 to 19.3%).

**Table 1:** Demographics of study population by SGA status.

	AGA (n = 300)	SGA (n = 43)	*p* value
**Gestational age (weeks)**			
Mean ± SD	28.43 ± 2.67	29.89 ± 3.19	0.0013
(Range)	(22.14–34.43)	(22.43–34.29)	
**Birth weight (g)**			
Mean ± SD	1172.5 ± 403.4	899.1 ± 291	< 0.0001
(Range)	(470–2940)	(410–1440)	
**No. (%) of infants inborn**	143 (47.67%)	23 (53.49%)	0.475
**No. (%) of male infants**	159 (53%)	31 (72.09%)	0.019
**CGA at worst exam (weeks)**			
Mean ± SD	35.81 ± 2.99	37.16 ± 3.92	0.038
(Range)	(30.71–52.29)	(31.43–49.14)	
**DOL at worst ROP exam (days)**	50.62 ± 29.18	48.41 ± 34.37	0.822
**Weight z-score change from birth to worst ROP exam **^**a**^	− 1.52 ± 0.82 (n = 244)	− 1.39 ± 1.14 (n = 39)	0.504

^a^Indicates smaller sample sizes due to missing data. Sample sizes used for statistical analyses are noted in each column.

**Table 2:** Demographics of study population by IUGR status.

	Non-IUGR (n = 257)	IUGR (n = 86)	*p* value
**Gestational age (weeks)**			
Mean ± SD	29.37 ± 2.36	26.35 ± 2.69	< 0.0001
(Range)	(22.86–34.43)	(22.14–33.14)	
**Birth weight (g)**			
Mean ± SD	1246.5 ± 369.8	814.5 ± 306.5	< 0.0001
(Range)	(410–2940)	(410–2245)	
**No. (%) of infants inborn**	129 (50.19%)	37 (43.02%)	0.249
**No. (%) of male infants**	146 (56.81%)	44 (51.16%)	0.362
**CGA at worst exam (weeks)**			
Mean SD	35.83 ± 2.96	36.44 ± 3.63	0.167
(Range)	(31.14–52.29)	(30.71–49.14)	
**DOL at worst ROP exam (days)**	45.20 ± 25.40	70.62 ± 33.75	< 0.0001
**Weight z-score change from birth to worst ROP exam**^**a**^	− 1.47 ± 0.83 (n = 212)	− 1.60 ± 0.90 (n = 71)	0.296

^a^Indicates smaller sample sizes due to missing data. Sample sizes used for statistical analyses are noted in each column.

In this cohort, 129 patients (38%) had CLD. There was no significant difference in CLD frequency when comparing SGA to AGA (AGA: 37.79%, SGA: 37.21%; *p* = 0.941, 95% CI − 16.0 to 14.9%), but IUGR infants were more likely to have CLD than non-IUGR infants (non-IUGR: 29.53%, IUGR: 61.36%; *p* < 0.0001, 95% CI − 43.4 to − 20.2%). 46% of infants had postnatal growth failure. Neonates with postnatal growth failure had an average weight gain of 98.3 g/week, compared to 137.7 g/week in infants without postnatal growth failure. There were 11 deaths before discharge with 5/11 being non-IUGR and AGA, 2/11 being SGA. 3/11 being IUGR and 1/11 being categorized as both SGA and IUGR. Neither SGA or IUGR status were significantly associated with death (AGA: 1.67%, SGA: 6.98%; *p* = 0.226, 95% CI − 2.4 to 13.1%) (non-IUGR: 1.96%, IUGR: 4.55%; *p* = 0.735, 95% CI − 2.1 to 7.3%).

### SGA versus AGA infants

**Figure 2:**
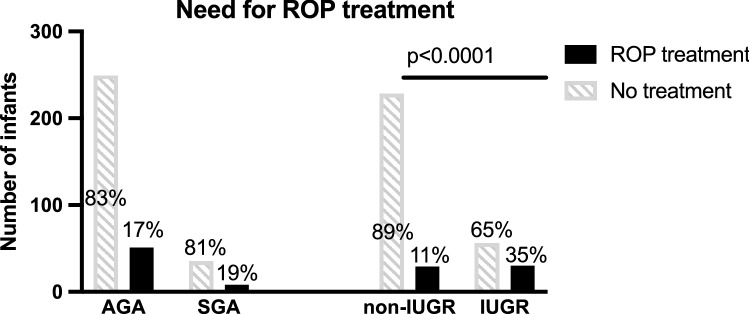
IUGR infants, but not SGA infants, are more likely to require treatment for ROP. There was no significant association observed between the need for ROP treatment and SGA (18.6%) versus AGA (17%) status (*p* = 0.794 by Chi-square). In the IUGR group, 34.9% of infants (30/86) were treated for severe ROP while the infants without growth restriction were treated at a rate of 11.3% (29/257) (*p* < 0.0001 by Chi-square)**.**

**Figure 3:**
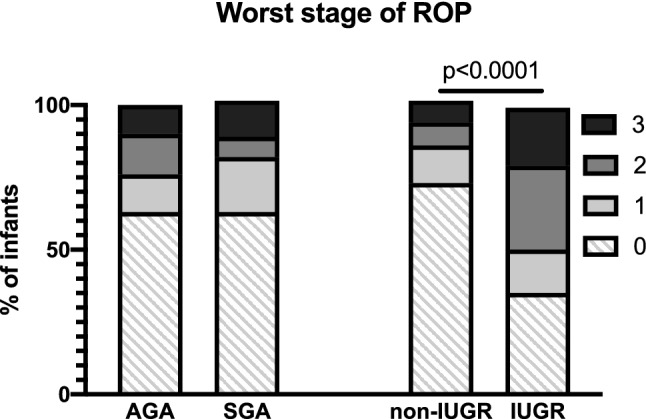
IUGR infants, but not SGA infants have more severe ROP. No significant association was observed between the worst stage of ROP and SGA status (AGA n = 300, SGA n = 43; *p* = 0.879 by Chi-square). However, there was a significant association observed between the worst stage of ROP and IUGR (non-IUGR n = 257, IUGR n = 86; *p* < 0.0001 by Chi-square) with a higher proportion of IUGR infants than non-IUGR infants in stage 1 (non-IUGR: 13.33%, IUGR: 14.77%), stage 2 (non-IUGR: 7.78%, IUGR: 29.07%) and stage 3 disease (non-IUGR: 6.61%, IUGR: 19.77%), and a higher proportion of non-IUGR infants with no ROP (stage 0) than IUGR infants (non-IUGR: 72.94%, IUGR: 35.23%).

**Figure 4:**
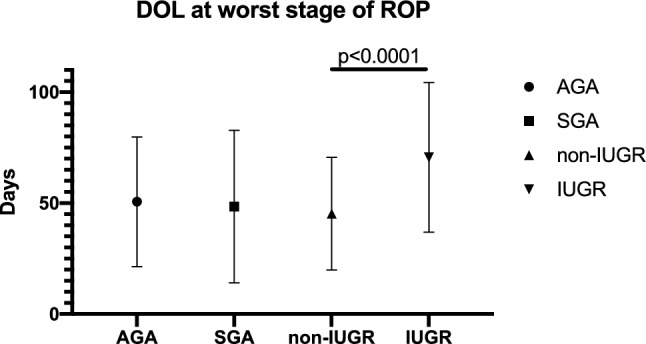
IUGR infants are older when they exhibit worse ROP disease than non IUGR infants. There was no statistically significant difference between SGA and AGA when comparing days of life (DOL) at worst stage of disease (AGA n = 300, SGA n = 43; *p* = 0.822 by a two-tailed t-test). There was a statistically significant difference between IUGR and non-IUGR infants in DOL at worst stage of disease, with IUGR infants being relatively older than non-IUGR infants (non-IUGR n = 256, IUGR n = 86; *p* < 0.0001 by two-tailed t-test). Symbols represent means and error bars indicate standard deviation.

In the SGA group, 18.6% of infants (8/43) had ROP disease severe enough to require treatment while in the AGA group 17.0% of infants were also treated (51/300) (*p* = 0.794, 95% CI − 10.8–14.0%) (Fig. 2). In addition, no significant difference was observed in the worst stage of ROP between SGA and AGA infants (*p* = 0.489, difference = 0.17, 95% CI − 0.25 to 0.43) (Fig. 3). In the SGA group, certain patterns emerged. The SGA babies had smaller BWs (AGA mean: 1172.5 ± 403.4 g, SGA mean: 899.1 ± 291; *p* < 0.0001), as expected, and higher GAs at birth (AGA: 28.42 ± 2.67, SGA: 29.89 ± 3.19; *p* = 0.0013) than their AGA counterparts (Table 1). However, there was not a significant interaction between birthweight or GA in SGA infants with worst stage of ROP (*p* = 0.196, *p* = 0.120, respectively) or need for treatment (*p* = 0.882, *p* = 0.339). There was no difference in weight z-score change from birth to time of worst ROP stage in AGA compared to SGA infants (AGA: − 1.52 ± 0.82, SGA: − 1.39 ± 1.14; *p* = 0.511 by t-test), and no significant difference in the proportion of SGA versus AGA infants with growth failure at the time of worst ROP exam (AGA: 47.5%, SGA: 35.9%; *p* = 0.176 by Chi-square, 95% CI − 4.7 to 27.9%). The CGA at worse exam was 9.4 days later in SGA infants than AGA infants (AGA: 35.81 ± 2.99 weeks, SGA: 37.16 ± 3.19; *p* = 0.038). However, given that the SGA cohort GA at birth was 10.1 days later than AGA babies, we compared day of life at which worst ROP stage occurred and there was no difference between SGA and AGA groups (AGA: 50.62 ± 29.18, SGA: 48.41 ± 34.37; *p* = 0.822 by t-test) (Fig. 4). However, infants with postnatal growth failure (regardless of SGA versus AGA) had worse stages of ROP (by 0.58; *p* < 0.001, 95% CI 0.35–0.81). In our cohort, when adjusting for SGA status, infants with growth failure were 1.8 times more likely to need treatment than infants without growth failure, though this did not reach statistical significance (OR: 1.85, 95% CI 0.97–3.52, *p* = 0.061).

### IUGR versus non-IUGR infants

The demographic characteristics (GA, BW, and CGA at worst ROP exam) for the IUGR group are shown below (Table 2). The etiologies for IUGR were: multiple gestation pregnancy (57.0%), maternal hypertensive disorder (including gestational hypertension, preeclampsia, HELLP syndrome) (34.9%), maternal hematologic disorder (i.e. prothrombotic disorder) (5.8%), and idiopathic (no other risk factor identified) (18.6%). In 14 patients, the mother had two potential risk factors (11 had both multiple gestation and hypertensive disorders and 3 had both multiple gestation and a maternal hematologic disorder). There was no significant association between worst stage of ROP, or need for ROP treatment, with any of these specific etiologies.

**Figure 5:**
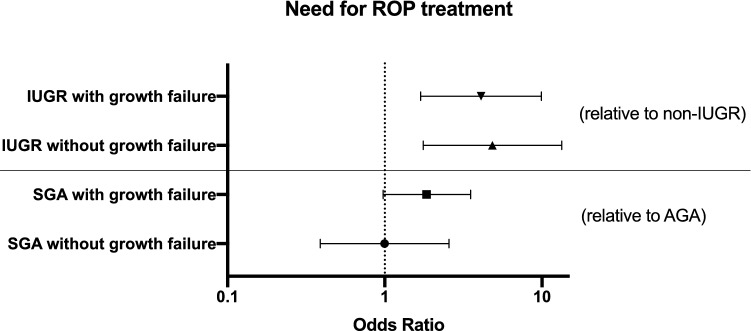
IUGR status, regardless of growth failure or not, is associated with increased need for ROP treatment. SGA infants with postnatal growth failure have increased risk for needing ROP treatment, though this was not significant when compared to AGA infants with postnatal growth failure (*p* = 0.06). IUGR infants with or without growth failure have a 4–5 times increased risk for needing ROP treatment compared to non-IUGR infants with or without growth failure (*p* < 0.0001). Of note, these comparisons were adjusted for growth failure status alone, not GA or BW at birth. Symbols and error bars represent odds ratio and 95% confidence intervals.

ROP stage was significantly worse in IUGR infants (by 0.84, 95% CI 0.57–1.11, *p* < 0.001 by t-test) compared to non-IUGR infants. There was a higher proportion of IUGR infants than non-IUGR infants in stage 1 (non-IUGR: 13.33%, IUGR: 14.77%), stage 2 (non-IUGR: 7.78%, IUGR: 29.07%) and stage 3 disease (non-IUGR: 6.61%, IUGR: 19.77%), and a higher proportion of non-IUGR infants with no ROP (stage 0) than IUGR infants (non-IUGR: 72.94%, IUGR: 35.23%) (Fig. 3). In the IUGR group, 34.88% of infants (30/86) were treated for severe ROP while the infants without growth restriction were treated at a rate of 11.3% (29/257) (*p* < 0.0001, 95% CI 13.4–35.0%) (Fig. 2). The IUGR babies had smaller BWs (non-IUGR: 1246.5 ± 369.8 g, IUGR: 814.5 ± 306.5 g; *p* < 0.0001), as expected, and lower GAs at birth (non-IUGR: 29.0 ± 2.4 weeks, IUGR: 25.9 ± 2.7 weeks; *p* < 0.0001) than their non-IUGR counterparts (Table 2) in this cohort. However, there was a significant interaction between birthweight and GA in IUGR infants with worst stage of ROP (*p* = 0.0007, *p* = 0.026) but not need for treatment (*p* = 0.382, *p* = 0.957, respectively). When accounting for the difference in BW, IUGR was no longer associated with need for ROP treatment (OR = 1.33, 95% CI 0.65–2.73, *p* = 0.436). However, IUGR was still associated with worse ROP stage by 3.12 (95% CI 2.58–3.71; *p* < 0.0001). When accounting for the difference in GA at birth, IUGR was no longer associated with need for ROP treatment (OR = 1.31, 95% CI 0.64–2.72, *p* = 0.462). However, IUGR was still associated with worse ROP stage by 2.42 (95% CI 0.42–4.42; *p* = 0.018). There was also a significant interaction between IUGR status and GA at birth, such that for every 1 week increase in GA at birth, the worse stage decreased by 0.21 in non-IUGR infants (95% CI 0.17–0.25, *p* < 0.001) and by 0.29 in IUGR infants (95% CI = 0.22–0.36; *p* < 0.001). There was a difference in the proportion of IUGR versus non-IUGR infants who exhibited growth failure at worst ROP stage (non-IUGR: 42.0%, IUGR: 57.8%, *p* = 0.021 by Chi-square, 95% CI4.2–30.7%). Adjusting for IUGR status, those with growth failure had worse ROP stage by 0.48 (95% CI 0.26–0.69, *p* < 0.001). Regardless of growth failure status, IUGR infants were 4–5 times more likely to need treatment for ROP (IUGR without growth failure: OR = 4.84, 95% CI = 1.76–13.35; IUGR with growth failure: OR = 4.09, 95% CI = 1.69–9.91) (Fig. 5). The CGA for IUGR infants at worst stage of disease was 4.5 days later than non-IUGR infants, which was not statistically significant (non-IUGR: 35.8 ± 3.0wks, IUGR: 36.4 ± 3.6; *p* = 0.167), despite lower GAs at birth (with IUGR infants born an average of 21 days earlier than non-IUGR infants). However, the DOL at worst stage of ROP for IUGR infants was 25.4 days later, on average, than in non-IUGR infants (non-IUGR: 45.2 ± 25.4 days, IUGR: 70.6 ± 33.8 days; *p* < 0.0001 by t-test), indicating that IUGR infants developed worse ROP at a relatively later postnatal age than non-IUGR infants (Fig. 4).

### IUGR versus SGA infants

**Figure 6:**
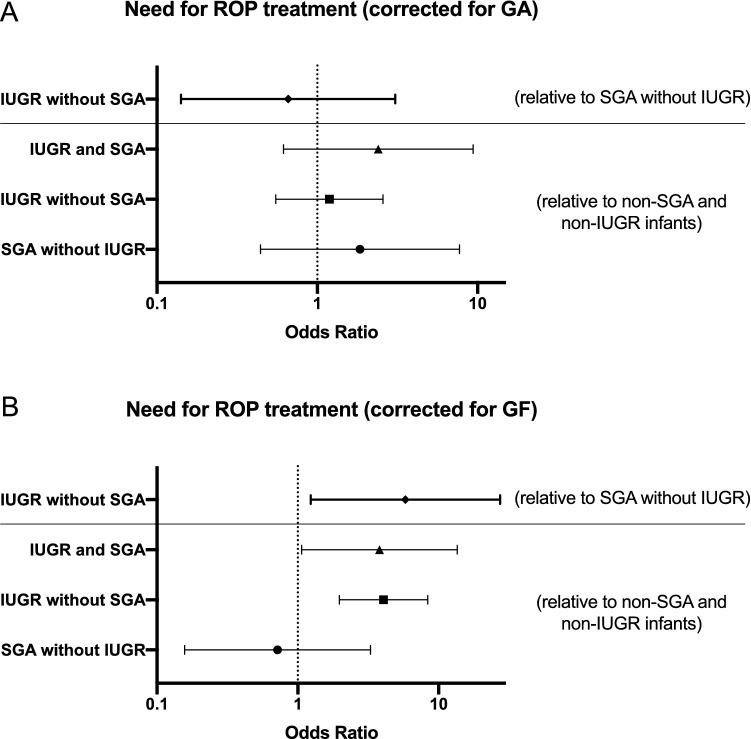
Infants with IUGR are more likely to need ROP treatment than SGA infants, regardless of postnatal growth failure status. When directly comparing IUGR infants (without SGA) to SGA infants (without IUGR), IUGR infants with or without growth failure have a 5.8 × increased risk for needing ROP treatment compared to SGA infants with or without growth failure (panel A, top line). Of note, when correcting for GA at birth, there is no statistically significant increased risk for need for ROP treatment in IUGR infants (without SGA) compared to infants with SGA (without IUGR) (*p* = 0.59) (panel B, top line). Also depicted in both graphs are comparisons of SGA infants without IUGR, IUGR without SGA, and infants with IUGR and SGA to infants without SGA status or IUGR (second, third and fourth lines, respectively).

When directly comparing whether IUGR differentially affects risk of ROP severity compared to SGA status, we compared only mutually exclusive groups, to test whether IUGR infants that are not SGA (n = 72) are at increased risk for worse ROP compared to SGA infants who were not IUGR (n = 29). When comparing IUGR infants (without SGA) to SGA infants (without IUGR), adjusting for GA, there were no differences in worst stage of disease (*p* = 0.095) or need for treatment (*p* = 0.595) (Fig. 6A) and there was not a significant interaction between mutually exclusive IUGR or SGA and GA for worst stage (*p* = 0.382) or need for treatment (*p* = 0.518). When comparing IUGR infants (without SGA) to SGA infants (without IUGR), adjusting for BW, IUGR infants had worse stages of ROP (by 0.69 ± 0.186; *p* = 0.0003) but not need for treatment (*p* = 0.114) and there was a significant interaction between mutually exclusive IUGR or SGA and BW for worst stage (*p* = 0.006) but not need for treatment (*p* = 0.484). IUGR infants without SGA were more likely to have growth failure than SGA infants without IUGR (61.02% versus 34.62%; *p* = 0.025 by Chi-square testing). IUGR infants with growth failure gained, on average, 45.1grams less per week than IUGR infants without growth failure (no GF: 147.9 g/week versus GF: 102.8 g/week). Similarly, SGA infants with growth failure gained, on average, 46.8 g/week less than SGA infants without growth failure (no GF:146.7 g/week versus GF: 99.9 g/week). When correcting for growth failure, IUGR infants had worse stages of ROP (by 0.793 ± 0.216; *p* = 0.0003) and increased need for ROP treatment (OR = 5.809, 95% CI 1.236–27.313; *p* = 0.026) compared to SGA infants without IUGR (Fig. 6B).

We also compared SGA without IUGR (n = 29), IUGR without SGA (n = 72), and infants with IUGR and SGA (n = 14) to infants without SGA or IUGR (n = 228). As you move from infants without SGA or IUGR to infants with SGA only to infants with IUGR only to infants with IUGR and SGA, the worst stage of disease increases (*p* < 0.0001 by Chi-square) and the need for treatment increases (*p* < 0.0001 by Chi-square). When correcting for GA alone, there were not significant differences in worst stage of ROP or need for treatment when comparing IUGR groups to non-SGA/non-IUGR (Fig. 6A). When correcting for BW alone, there were not significant differences in worst stage of ROP (*p* = 0.342) or need for treatment (*p* = 0.766) when comparing IUGR groups to non-SGA/non-IUGR. When correcting for growth failure alone, IUGR infants (without SGA) had significantly worse stage of disease (by 0.77) than infants without SGA or IUGR (*p* < 0.0001) and no other comparisons were significant. When correcting for growth failure alone, IUGR infants without and with SGA had a 3–4 times increased need for treatment (IUGR without SGA: OR 4.064, 95% CI 1.974–8.364, *p* = 0.0001; IUGR with SGA: OR 3.802, 95% CI 1.074–13.581, *p* = 0.040) compared to infants without IUGR or SGA (Fig. 6B).

## Discussion

While this study found an association between prenatal IUGR and worse ROP, it did not find an association between SGA and ROP. In the literature, there appears to be a lack of consensus as to whether or not SGA is directly associated with ROP^7–12^. A recent meta-analysis found a positive association between SGA status and severe and treated ROP^7^, while another study found a relationship between poor postnatal weight gain and ROP, but no association with SGA and ROP^11^. A major contributor to the discrepancy between these studies is the variable definitions for SGA, which has been shown to greatly reduce the ability to draw consistent conclusions about outcomes for this cohort^13^. In various studies, SGA and growth restriction were used interchangeably, and defined with various BW percentile cut-offs.

To our knowledge the relationship between prenatal IUGR and ROP has not been previously studied, and thus our study is unique and impactful in this respect. Specifically, we found that, IUGR but not SGA alone increased risk of worse stage of ROP and need for ROP treatment, supporting our hypothesis that these are pathologically distinct conditions that affect postnatal development in premature infants. In addition, this study separated worst stage of ROP, need for ROP treatment, and DOL at worst severity as outcomes. When we corrected for the lower GA of the IUGR cohort, we no longer found a significant association with need for treatment, but the association of IUGR with worst stage of ROP remained. It is known and well-established that older GA at birth is a protective factor against ROP, whether a preterm infant is IUGR or not, which we also demonstrated in our study (non-IUGR infants show a decrease in worst stage of ROP by 0.21 with each additional week of GA at birth). Interestingly, we found that higher GA at birth protected against ROP more in IUGR infants than non-IUGR infants suggesting the benefits of delaying preterm delivery specific to ROP risk is even greater in IUGR infants. Moreover, we found that IUGR infants had worst ROP stage significantly later than non-IUGR infants which emphasizes the importance of longer-term follow-up beyond an infant’s due date.

Poor postnatal weight gain, and low IGF-1 levels as a surrogate, have been associated with increased risk of treatment-requiring ROP^14–16^. While prior studies sought an absolute amount of weight gain over a specified period in an effort to eliminate screening eye exams, the purpose of our study was not to reduce eye exams, but to investigate the pathophysiologic effect of prenatal and postnatal weight gain in ROP development. Therefore, just as IUGR is defined as a change in prenatal weight gain over time, we similarly defined failure of postnatal weight gain as a significant decline in z-score between birth and the time of worst ROP stage, capturing the time period of most relevance. Postnatal growth failure, like IUGR, has been variably defined and drawing conclusions across studies who define postnatal growth failure differently is problematic^17^. In general, postnatal growth failure is defined as the inability to achieve in-utero growth rate (and body composition) for a fetus of the same GA. However, the specifics of how this is defined varies by study, though the most well-accepted method to evaluate quality of nutritional care utilizes a decrease in weight z-score over a pre-defined period of time^17,18^. Nevertheless, impaired fetal and postnatal growth have both been associated with worse outcomes, including neurodevelopmental impairment and cardiometabolic disease, and is therefore an important factor to consider when evaluating neonatal outcomes such as ROP^19,20^.

In our cohort, IUGR infants were at greater risk for postnatal growth failure, a known potential co-morbidity of IUGR^5,21^ and we also found that postnatal growth failure, independent of growth status at birth, increased risk of worse ROP disease. On average, infants with growth failure, regardless of their SGA or IUGR status gained ~ 40 g/week less than their counterparts without growth failure in our cohort. Importantly, we found that IUGR infants with or without postnatal growth failure all have a 4–5 × increased need for treatment for ROP, suggesting that the pathologic prenatal environment in IUGR is an independent risk factor for worse ROP, separate from postnatal growth.

To directly compare IUGR to SGA, we separately compared mutually exclusive groups only—SGA without IUGR to IUGR without SGA to neither IUGR or SGA to both IUGR and SGA infants. We demonstrate that over the progression of potential prenatal growth impairment—from no growth impairment to the most severe IUGR (IUGR and SGA infants)— the worst stage of ROP and need for treatment for ROP increases. When correcting for GA, we were no longer powered to detect a difference between IUGR only (without SGA) and SGA only (without IUGR). This may suggest that the tendency for IUGR infants to be born more prematurely is a major player in the pathomechanism for greater propensity towards worse ROP disease in IUGR. Importantly, when we corrected for birthweight, IUGR without SGA infants still have worse stages of ROP but not increased need for ROP treatment (*p* = 0.11), though our inability to detect differences in need for treatment may have again been hindered by small sample sizes in these mutually exclusive groups. Larger studies will need to be conducted in order to validate whether IUGR is mechanistically a separate pathologic driver mediating ROP risk, and not just a proxy for low birth weight. Interestingly, when correcting for postnatal growth failure, we still found that regardless of growth failure status, IUGR infants (without SGA) were more likely to need ROP treatment than SGA infants (without IUGR), suggesting that adequate postnatal growth does not counteract true pathologic prenatal growth impairment in terms of ROP outcome.

Limitations of this study includes the difficult categorization of infants as IUGR, which is difficult both in clinical practice and for research purposes. IUGR is a diagnosis that is at the discretion of the obstetrics team caring for the mother and fetus prenatally. The percentage of premature neonates identified as IUGR (88/343, 26%) in our study was slightly higher than in other study cohorts with reported ranges from ~ 10 to 20%, depending on race and developed vs. developing nations^22–25^, while our SGA cohort was comparable (53/343, 12.5%) to other reported cohorts. We believe that our IUGR incidence is higher than in other studies because our center serves as a referral high-risk obstetric center, with especially high rates of multiple-gestation pregnant mothers and pregnant women with gestational disorders including hypertension. One aspect that makes the diagnosis of IUGR difficult is that IUGR, especially idiopathic IUGR, may only become obvious in the third trimester. Our comparably high rate of IUGR may have allowed us to detect differences which other studies may not have been powered to do. However, when adjusting for potential important covariates such as GA in our observational study, we were no longer able to detect significant differences in ROP outcomes when using smaller sample sizes (comparisons among mutually exclusive SGA/IUGR status groups). Nonetheless, our study independently evaluated IUGR and SGA status, with the full recognition that there is overlap between these groups, and found that IUGR status, not SGA status, was associated with worse ROP outcomes. Another limitation of our study is that the cohort spans many years, during which there have been changes in neonatal practices, including target oxygen saturations, which certainly would have implications on ROP rates^26^. While the optimal target saturation to balance the reported increased risk of death when targeting lower saturations (85–89%) with the increased risk of ROP when targeting higher saturations (91–95%) remains debated, the main trial which reported these findings was published during the time period included in our study, which may have resulted in changes in practice which would result in lower rates of ROP that may have affected our study conclusions^27^.

In conclusion, our study is among the first to specifically distinguish altered prenatal growth patterns and low BW by focusing on IUGR versus SGA. We found that IUGR, and not SGA alone, was an independent risk factor for worse ROP. Lower GA at birth may be a significant factor in worse ROP outcomes in IUGR infants. We also found that increasing GA at birth is more protective against ROP in IUGR than non-IUGR infants. Lastly, we demonstrated that IUGR neonates have a significantly later DOL at worst stage of ROP, indicating that they need to be followed closely for the late development of treatment-requiring ROP compared to their non-IUGR counterparts. Avoidance of postnatal growth failure, while reported as protective in premature infants, did not appear protective in IUGR infants in our study, as risk of needing ROP treatment in IUGR infants was increased regardless of postnatal growth failure status. These findings add to the understanding of how prenatal environment affects risk of postnatal disease in premature infants.

## Methods

A retrospective chart review was conducted at a single level IV neonatal intensive care unit in Los Angeles, CA, USA (University of California-Los Angeles) on patients screened for ROP between January 1, 2011 and December 31, 2018. All methods were carried out in accordance with guidelines and regulations set forth by the UCLA Institutional Review Board.

### Participants

All infants hospitalized in the neonatal intensive care unit (NICU) who required screening examinations for ROP were eligible. At UCLA, inclusion criteria were consistent with AAP guidelines: infants born at a gestational age (GA) ≤ 30 weeks, BW < 1500 g, or GA at birth > 30 weeks but with an unstable clinical course. Exclusion criteria included any subject without prenatal records available to determine IUGR status, infants with IUGR due to congenital viral infections or genetic abnormalities, or missing data on ROP examination outcomes. The Institutional Review Board at UCLA approved the research protocol and waived the requirement of consent.

### Study protocol

Infants were identified by electronic medical record review and via a log of patients who received ROP screening after birth maintained by the Pediatric Ophthalmology team at UCLA. Patient demographic information, including gestational age (GA) at birth, sex, BW, corrected gestational age (CGA) at the time of worst ROP exam, days of life (DOL) at worst ROP exam, weight gained and weight at worst stage of ROP, diagnosis of chronic lung disease (CLD) (defined as need for supplemental oxygen or respiratory support at 36 weeks CGA), and death before hospital discharge was collected when available. SGA was defined as a BW percentile of < 10%^28^. IUGR was broadly diagnosed by the prenatal obstetric team by deceleration of fetal growth as determined by serial fetal ultrasound. Etiologies for IUGR were identified according to maternal risk factors including presence of gestational hypertensive disorders (including preeclampsia, gestational hypertension, or HELLP syndrome), maternal hematologic disorders leading to a pro-thrombotic state, multiple gestation, or idiopathic (no identified risk factors). Change in postnatal growth was quantified by calculating the z-score of each infant at birth and at worst stage of ROP^28^. Postnatal growth failure was defined as a difference in z-scores of > 1.5^29^. The average weekly weight gain for each infant was calculated by dividing the difference between birthweight and weight at worst stage of ROP by the number of postnatal days at worst stage of ROP, multiplied by 7.

### Outcomes measures

The primary outcome variable was worst stage of ROP as determined by indirect ophthalmoscopy. ROP zone, stage, and presence or absence of plus disease was documented according to the International Classification of ROP^30^.

The secondary outcome variables evaluated were ROP requiring treatment, day of life (DOL) at the time of worst exam, and postnatal growth failure. ROP treatments included anti-vascular endothelial growth factor injection, laser photocoagulation therapy, or both. Criteria for treatment-requiring ROP was acute Type 1 ROP and persistent Type 2 ROP beyond 52 weeks.

Recorded data measures included demographic information (GA at birth, BW, sex, corrected GA at the time of worst ROP exam, weight at the time of worst ROP exam), whether the infant was inborn or outborn, and co-morbidities (including diagnosis of CLD, death).

### Analyses

Continuous variables were compared using the two-sample t-test. Association and trend were analyzed for categorical variables using the Chi-square test (or Fisher’s exact test) and Cochran Armitage trend test respectively. In analyzing ROP severity and need for treatment, general linear model and logistic regression models were used, respectively, incorporating IUGR or SGA status as the main study variables, adjusting for growth failure or GA or BW. To do this, in each model, we tested for the possible interaction between the main study variable and covariates (specifically growth failure, GA, and BW). Considering the high correlation between these covariates, each variable was entered into the model separately. Using a model that includes both IUGR and SGA with their interaction, we compared SGA and IUGR to non-SGA and non-IUGR, but also compared SGA to IUGR directly. We also used general linear modeling and logistic regression models to test the association of different IUGR etiologies to worst stage of ROP and need for treatment respectively. In these models, possible interactions between the IUGR etiologies and between IUGR etiology and growth failure or GA were tested. 95% confidence intervals are reported for the difference in the proportions. A *p* value < 0.05 was considered statistically significant. All analyses were performed using SAS 9.4 (Cary, NC).

## Data Availability

The datasets generated during and/or analyzed during the current study are available from the corresponding author on reasonable request.
